# Integrated Transcriptomic and Biochemical Analyses Reveal Coordinated Suppression of ER-Associated Survival Signaling and Activation of ROS-Mediated Stress Responses by Oxidized Graphene Quantum Dots in Estrogen Receptor-Positive Breast Cancer

**DOI:** 10.3390/ijms27209211

**Published:** 2026-10-16

**Authors:** Wei-Chung Shia, Chien-Te Hsieh, Po-Chih Yang, Wan-Ling Chuang, Wan-Ting Shen

**Affiliations:** 1 Laboratory of Molecular and Surgical Research, Department of Research, Changhua Christian Hospital, Changhua 50050, Taiwan; 2 Department of Chemical Engineering and Materials Science, Yuan Ze University, Taoyuan 32003, Taiwan; cthsieh@saturn.yzu.edu.tw (C.-T.H.); pcyang@saturn.yzu.ed.tw (P.-C.Y.)

**Keywords:** oxidized graphene quantum dots, breast cancer, MCF-7, estrogen receptor, PI3K-AKT-FoxO, nascent protein synthesis, oxidative stress

## Abstract

Oxidized graphene quantum dots (o-GQDs) alter viability and cell-cycle progression in breast cancer cells, yet the cellular programs underlying these effects remain incompletely defined. We exposed MCF-7 cells to 10 mg/L o-GQD for 72 h and integrated RNA sequencing with quantitative protein-marker, ROS, antioxidant-intervention, proteasome-inhibition, and puromycin-labeling analyses from independent experiments. Transcriptomic analyses showed significant negative enrichment of proliferative and cell-cycle programs (E2F Targets, G2M Checkpoint, MYC Targets V1), with complementary KEGG/Reactome evidence of attenuated estrogen-associated programs. Quantitative Western blotting showed dose-dependent reductions in ERα (to 41.36 ± 13.73% at 10 mg/L), p-RB (to 8.31 ± 10.39%), and Survivin (to 23.97 ± 19.01%), with p-ERK/ERK attenuation and marked γ-H2AX accumulation (345.21 ± 161.39%). The ROS-positive fraction rose from 6.86% to 30.46%; antioxidant co-treatment failed to rescue the phenotype and MG132 did not consistently restore protein markers, arguing against oxidative stress or proteasomal degradation as standalone drivers. Reduced puromycin labeling added a protein-homeostasis readout consistent with reduced nascent protein synthesis. BT-474 and MDA-MB-231 cells showed weaker or different marker responses. Together, these findings define a coordinated MCF-7 response spanning estrogen-associated and cell-cycle programs, survival markers, protein homeostasis, and cell fate.

## 1. Introduction

Estrogen receptor-positive (ER-positive) breast cancer cells depend on estrogen receptor-associated transcriptional programs that cooperate with PI3K-AKT signaling to promote proliferation and survival, and crosstalk among these pathways contributes to tumor progression and endocrine-therapy resistance [1,2,3]. Cell-cycle progression, FOXO-regulated survival and apoptosis mediators, and protein-turnover systems act downstream of or in parallel with these growth signals, so perturbation of the interconnected network can produce linked transcriptional, protein-level, oxidative-stress, and cell-fate responses. Integrating these complementary evidence layers provides a framework for resolving how survival signaling and protein homeostasis change together.

Graphene quantum dots (GQDs) are graphene-derived nanoscale carbon materials whose optical properties and modifiable surfaces have motivated investigation in bioimaging, drug delivery, and photo-assisted cancer applications [4]. In cancer-cell studies, GQDs have also been used as biological perturbations because preparations can alter proliferation, cell-cycle progression, and apoptosis, as reported previously for breast-cancer cell models [5]. Their cellular effects are material- and model-specific: graphene-based nanomaterials have been linked to stress-responsive autophagy [6], and one GQD preparation in a gastric-cancer cell model was reported to activate autophagic flux and attenuate hydrogen-peroxide-associated oxidative injury [7]. The effects of GQD preparations on redox and protein-homeostasis responses are therefore formulation- and cell-context-dependent, and the specific o-GQD preparation examined here is treated as a defined cellular perturbation rather than a receptor-targeted therapeutic with an established molecular target.

How these processes converge in breast-cancer cells remain poorly resolved. Estrogen receptors and the ubiquitin-proteasome system regulate each other [8], and graphene-based materials can engage redox- and autophagy-associated stress responses [6,7], yet the relationship between estrogen-associated signaling attenuation, protein-synthesis changes, and oxidative-stress responses during exposure to a defined GQD preparation has not been established. In prior work, we reported that a defined o-GQD preparation induced reduced viability, G2/M arrest, and apoptosis-related changes in breast cancer cell models [5]. Here, we define the integrated MCF-7 response across transcriptomic, ER-associated/cell-cycle, protein-homeostasis, oxidative-stress, and cell-fate evidence layers. We combined the pathway-level interpretation of RNA sequencing with quantitative protein-marker assessment and puromycin labeling. Antioxidant intervention and proteasome inhibition tested whether oxidative stress or enhanced protein degradation could sufficiently explain the broader response.

## 2. Results

### 2.1. MCF-7 Cells Showed the Strongest Response Among the Tested Models

To select a principal model for mechanistic analysis, we compared the viability responses of MCF-7, BT-474, and MDA-MB-231 cells to the PEGylated o-GQD preparation. We previously observed a dose-associated reduction in MCF-7 viability [5], whereas BT-474 and MDA-MB-231 showed smaller responses under the compared conditions. On this basis, MCF-7 was selected for the integrated mechanistic experiments, with the other cell lines providing a comparison context.

### 2.2. RNA Sequencing at 72 h Identified Cell-Cycle, Estrogen-Associated, and RNA-Processing Programs

**Figure 1:**
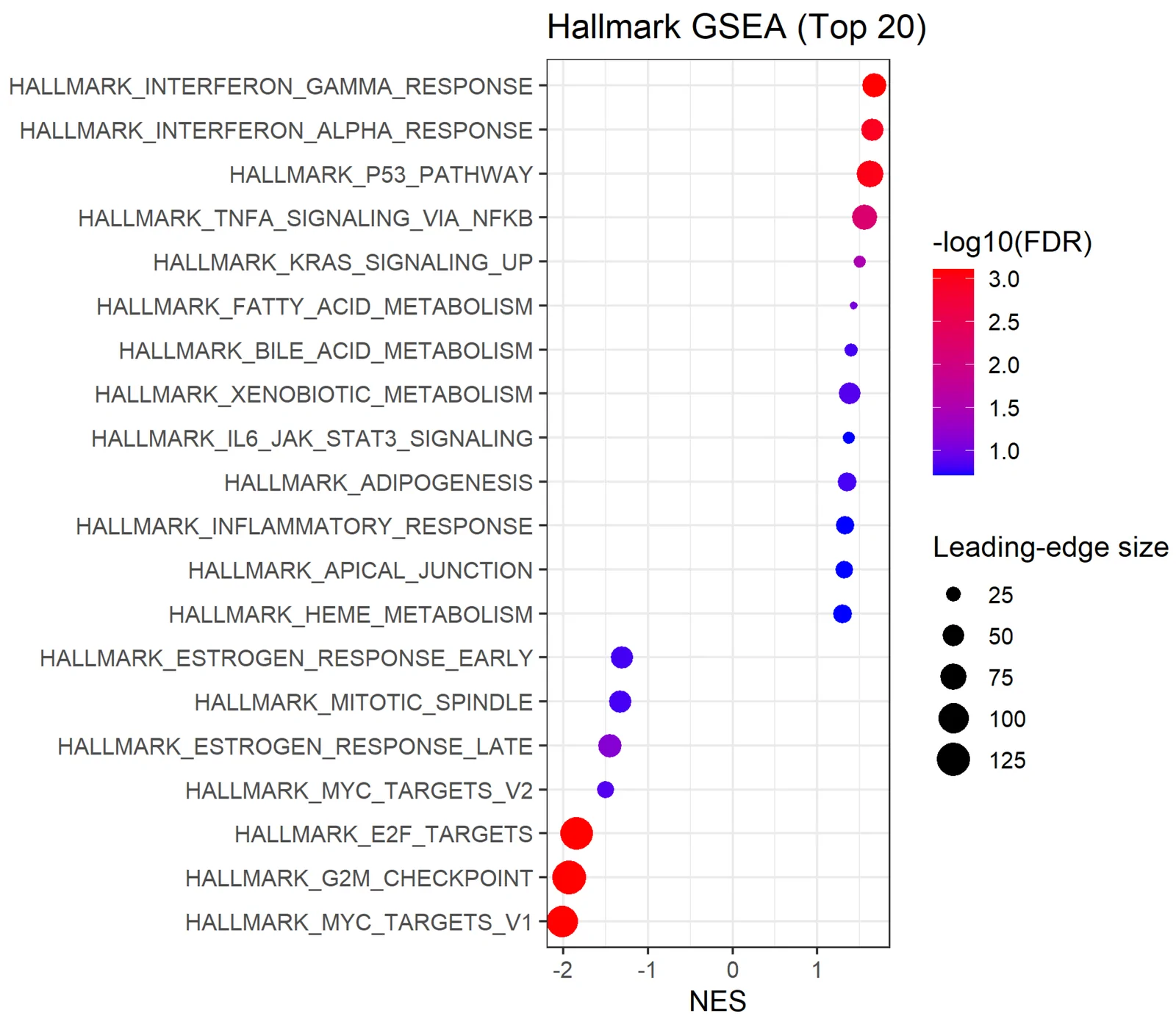
Transcriptomic programs after 10 mg/L o-GQD exposure for 72 h in MCF-7 cells. RNA sequencing compared MCF-7 cells exposed to 10 mg/L o-GQD for 72 h with time-matched untreated controls using three independent biological replicates per group. Ranked-list analyses showed negative enrichment of Hallmark E2F Targets, G2M Checkpoint, and MYC Targets V1.

We characterized the well-developed transcriptomic response at the selected 72 h endpoint by Hallmark GSEA and complementary GO, KEGG, and Reactome analyses of the RNA-sequencing data (Figure 1). Hallmark E2F Targets, G2M Checkpoint, and MYC Targets V1 showed significant negative enrichment, consistent with attenuation of proliferative and cell-cycle programs. Estrogen-associated evidence came from complementary pathway analyses: KEGG estrogen signaling and Reactome estrogen-dependent gene expression were negatively enriched, along with attenuation of chromosome-segregation, DNA-replication, and RNA-processing programs. The exact normalized enrichment scores, nominal *p* values, FDR values, and leading-edge genes for the Hallmark estrogen-response sets are reported in Supplementary Table S1; both sets reached nominal *p* < 0.05 with FDR ≥ 0.05 and are interpreted as directional rather than FDR-significant. Positive enrichment of p53-, interferon-, TNFα/NF-κB-, and KRAS-related Hallmark programs placed these changes in a stress-response context. These program-level findings complement the protein-marker response measured by Western blotting.

### 2.3. ROS Increased Alongside the Cellular Response; Antioxidant Co-Treatment Did Not Rescue the Phenotype

**Figure 2:**
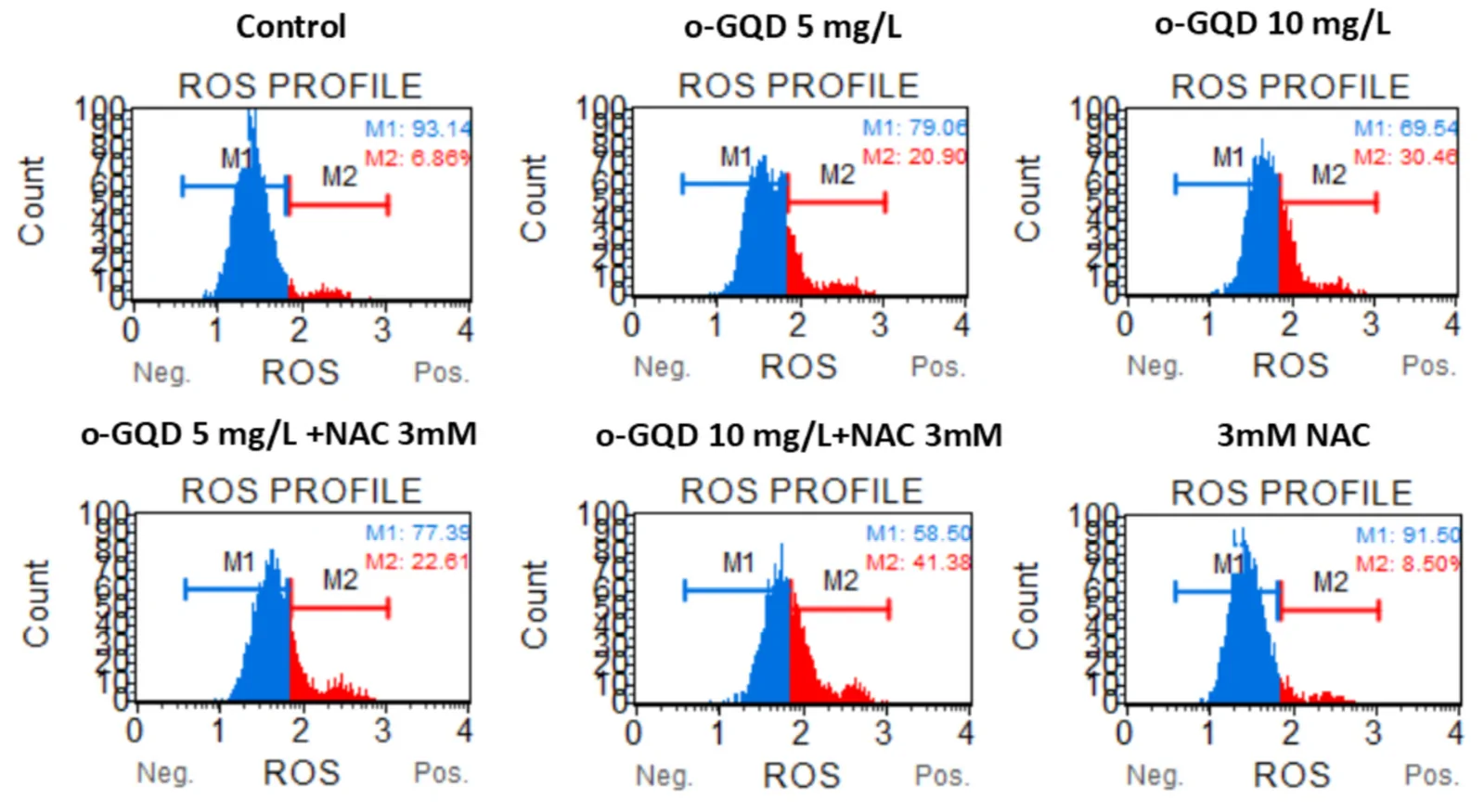
ROS response and antioxidant intervention in MCF-7 cells. Representative Muse Cell Analyzer histograms show ROS-positive and ROS-negative populations after treatment with 5 or 10 mg/L o-GQD for 72 h, with or without 3 mM NAC. We observed an increased ROS-positive fraction after o-GQD exposure, whereas NAC did not rescue the morphology or viability phenotype under the tested conditions.

We measured ROS and tested antioxidant interventions to assess the contribution of oxidative stress to the cytotoxic response (Figure 2). The ROS-positive fraction increased from 6.86% in control cells to 20.9% and 30.46% after treatment with 5 and 10 mg/L o-GQD, respectively. NAC or MitoTEMPO did not restore the morphology or viability phenotype under the tested conditions, and NAC did not consistently reduce the ROS-positive fraction in the displayed comparisons. Thus, ROS accompanied the response, while antioxidant co-treatment did not reverse the phenotype.

Supplementary Figure S1 extends the ROS analysis to 24 h, with ROS-positive and ROS-negative populations measured after exposure to 5 and 10 mg/L o-GQD, with or without 20 μM MitoTEMPO. A focused ROS/Annexin V time-course series in *n* = 2 independent experiments provides additional temporal context (Supplementary Figure S2); both experiments are shown descriptively.

### 2.4. Quantitative Western-Blot Analysis Supports ERα Reduction, RB Dephosphorylation, and Selected Survival/Stress Marker Changes in MCF-7 Cells

**Figure 3:**
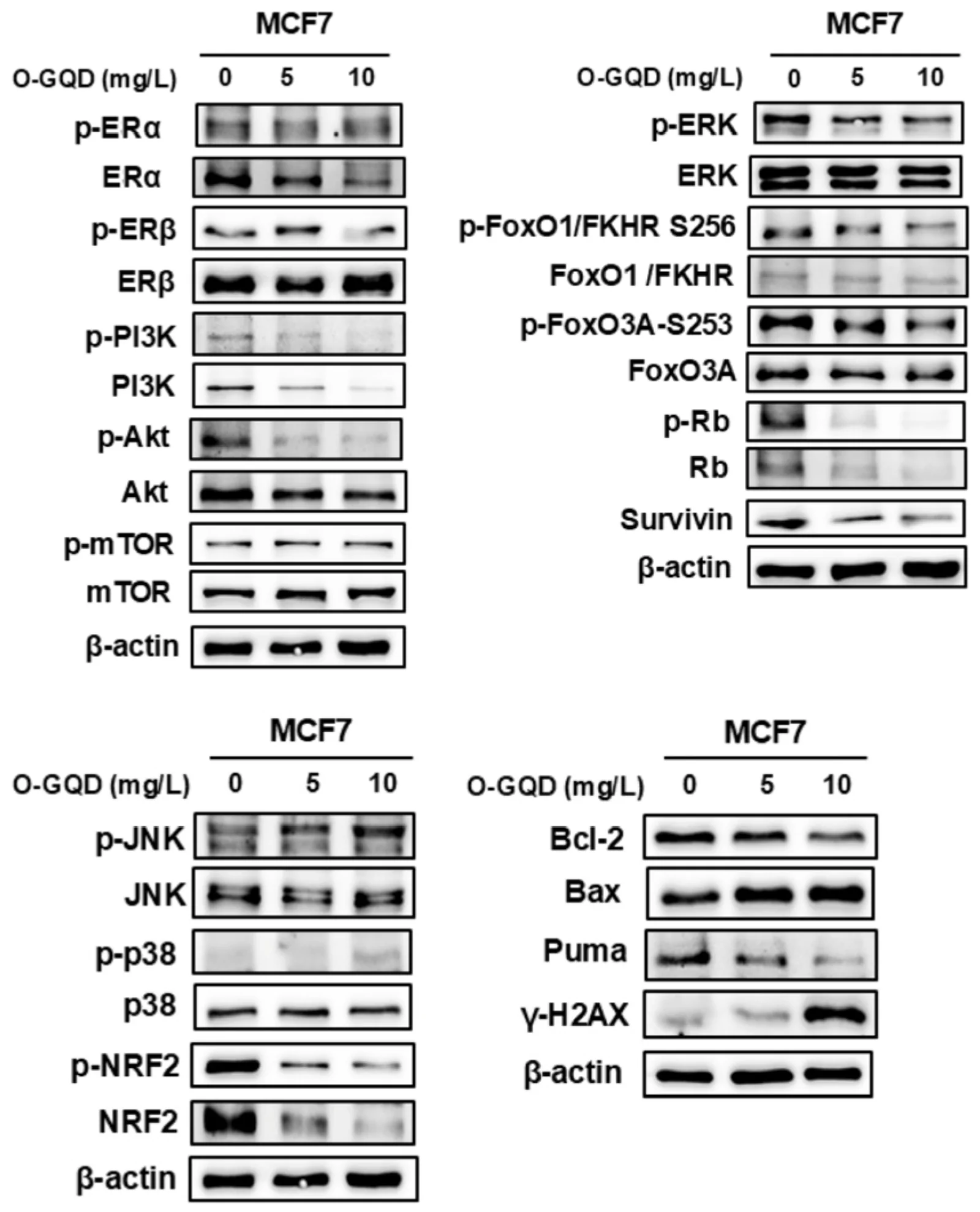
Western-blot analysis of ER-associated, FoxO/cell-cycle, survival, and stress-associated protein markers in MCF-7 cells after 72 h of o-GQD exposure. Quantification uses values normalized within each experiment to the untreated control; biological n varies by marker (generally n = 3, with n = 4 or 5 for selected endpoints). Endpoint-specific one-way repeated-measures ANOVA was used as the primary analysis, with Holm adjustment for the two matched dose-versus-control contrasts within each endpoint. Across-endpoint multiplicity adjustment was retained only as a conservative sensitivity analysis. Direct protein abundance and phospho/total ratios were analyzed separately. The strongest quantitative effects included p-RB, ERα, Survivin, p-ERK/ERK, p-FoxO1/FKHR-S256, and FoxO3A; γ-H2AX showed a marked dose-associated increase.

To examine protein-marker responses in MCF-7 cells, we assessed ER-associated, FoxO/cell-cycle, stress-, DNA-damage-associated-, and apoptosis-related markers after 72 h of o-GQD exposure (Figure 3). Quantitative analysis used Western-blot values normalized within each experiment to the untreated control and analyzed according to the matched experimental design. Direct ERα abundance decreased from the within-experiment control value of 100% to 61.59 ± 15.68% at 5 mg/L and 41.36 ± 13.73% at 10 mg/L (one-way repeated-measures ANOVA F(2,4) = 28.5947, *p* = 0.004273; Holm-adjusted 10-versus-0 *p* = 0.035592). In contrast, the p-ERα/ERα ratio itself was variable because both phosphorylated and total ERα decreased, emphasizing the distinction between protein abundance and phospho/total ratios.

Cell-cycle and survival markers showed some of the clearest quantitative effects. Direct p-RB abundance decreased to 13.40 ± 6.83% and 8.31 ± 10.39% at 5 and 10 mg/L, respectively (F(2,4) = 227.0947, *p* = 0.000076; Holm-adjusted *p* = 0.004131 and 0.004250). The p-RB/RB ratio likewise showed a strong overall treatment effect (F(2,4) = 57.4725, *p* = 0.001131). Survivin, quantified across four matched experiments, decreased to 44.66 ± 29.25% and 23.97 ± 19.01% (F(2,6) = 25.8008, *p* = 0.001130; Holm-adjusted *p* = 0.032350 and 0.008158). p-ERK/ERK also decreased consistently (74.23 ± 0.66% and 73.34 ± 10.48%; F(2,4) = 19.9658, *p* = 0.008290).

Within the FoxO-related readouts, p-FoxO1/FKHR-S256 abundance decreased to 49.83 ± 30.13% and 30.77 ± 18.47% (F(2,4) = 16.4099, *p* = 0.011802), while total FoxO3A decreased to 66.13 ± 21.61% and 42.24 ± 25.23% (F(2,4) = 12.3311, *p* = 0.019476). Other phospho/total ratios were more variable and were not interpreted as uniform pathway-wide suppression.

Among stress- and cell-fate-associated markers, γ-H2AX showed a marked dose-associated increase (129.07 ± 51.93% at 5 mg/L and 345.21 ± 161.39% at 10 mg/L; repeated-measures *p* = 0.049629), while BCL-2 showed a directional decrease at 10 mg/L (65.52 ± 22.22%; *p* = 0.073270) and BAX showed a directional increase (131.59 ± 36.41% at 10 mg/L; *p* = 0.168135). p-NRF2/NRF2 also showed a directional reduction, whereas PUMA and several PI3K/mTOR/JNK phospho/total ratios were variable across experiments.

Together, the quantitative findings identify strong RB dephosphorylation, reduced ERα abundance, Survivin suppression, selected FoxO/ERK marker changes, and accompanying γ-H2AX accumulation. These results provide targeted mechanistic evidence for selected components of the integrated MCF-7 response.

We also completed a focused Western-blot time course in two independent MCF-7 experiments using 10 mg/L o-GQD at 0, 24, 48, and 72 h (Supplementary Figure S3). Both experiments showed reduced ERα abundance by 24–48 h and progressive BCL-2 reduction, while BAX remained above baseline. Marked cleaved-PARP accumulation was observed at 72 h in both experiments. In contrast, p-AKT/AKT and p-FoxO3A-S253/FoxO3A showed non-monotonic or adaptive increases rather than uniform temporal suppression. Because this series comprised n = 2 biological experiments, both experiments are presented descriptively without formal inferential statistics; the series is not interpreted as establishing a complete causal chronology.

### 2.5. Comparison Cell Lines Showed Variable or Different Marker Responses

**Figure 4:**
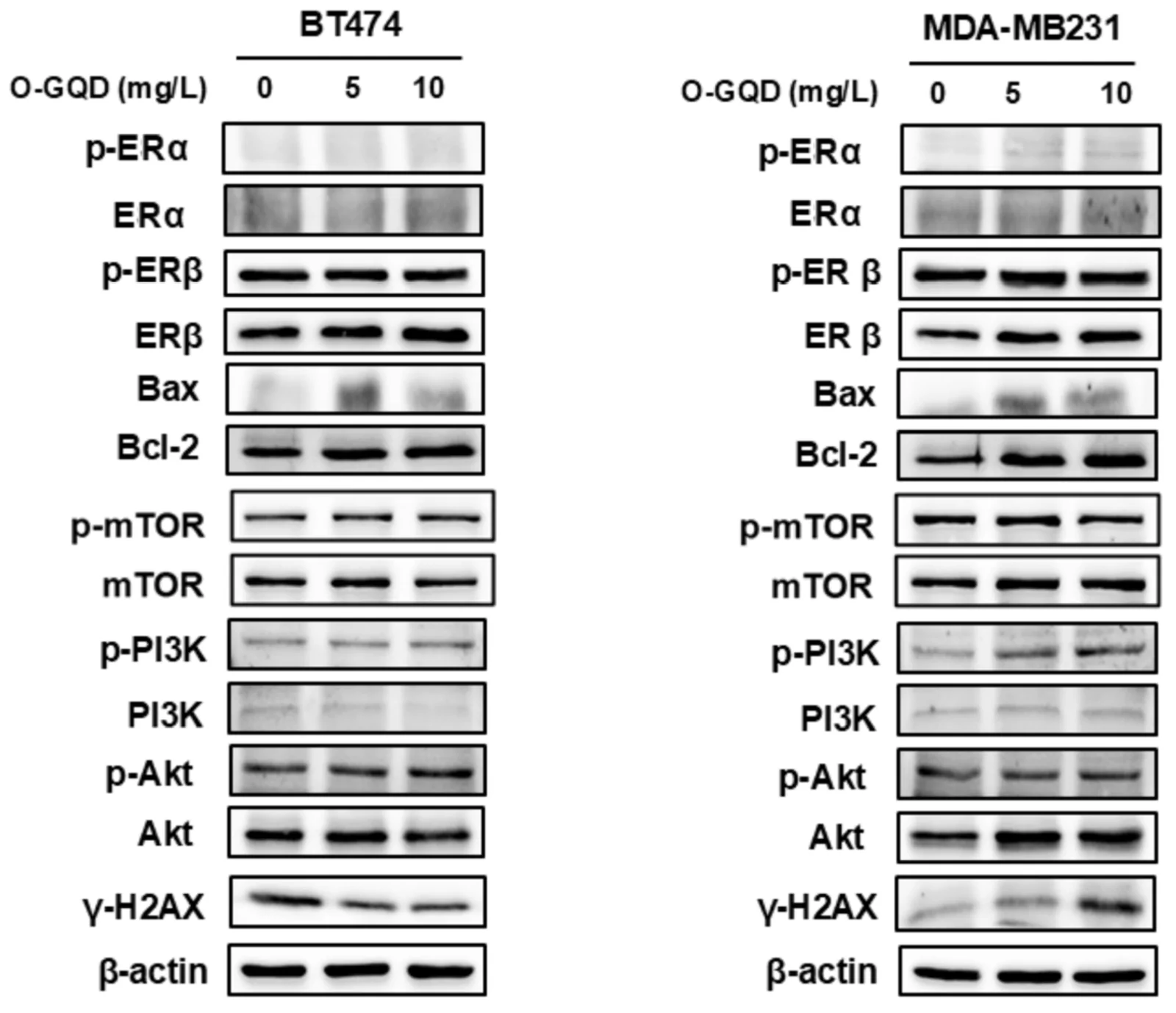
Comparison of selected signaling and stress-marker responses in BT-474 and MDA-MB-231 cells. Cells were treated with 0, 5, or 10 mg/L o-GQD for 72 h. Quantification is based on three independent biological experiments per cell line. Values are normalized to the within-experiment untreated control and are shown as mean ± SD with individual biological-replicate points. For each quantified marker, dose effects were assessed by one-way repeated-measures ANOVA across 0, 5, and 10 mg/L; matched 5-versus-0 and 10-versus-0 mg/L comparisons were Holm-adjusted when post hoc comparisons were examined. In BT-474 cells, no quantified marker showed a significant omnibus dose effect. In MDA-MB-231 cells, p-mTOR showed an omnibus dose effect (F(2,4) = 15.7149, *p* = 0.012746), but neither matched dose-versus-control comparison remained significant after Holm adjustment (adjusted *p* = 0.180667 and 0.203074). γ-H2AX showed directional accumulation in the MDA-MB-231 series, but the repeated-measures ANOVA did not reach significance (*p* = 0.124629).

To define the boundaries of the MCF-7-associated marker pattern, we quantified selected ER-, PI3K-AKT-mTOR-, BAX/BCL-2-, and γ-H2AX-related markers in BT-474 and MDA-MB-231 cells after exposure to 0, 5, or 10 mg/L o-GQD for 72 h using three independent biological experiments (Figure 4). In BT-474 cells, the quantified ER- and PI3K-AKT-related markers were variable across experiments, and no endpoint showed a significant omnibus dose effect by one-way repeated-measures ANOVA. In MDA-MB-231 cells, most quantified pathway markers likewise did not show a statistically stable dose–response pattern. An omnibus dose effect was detected for p-mTOR (F(2,4) = 15.7149, *p* = 0.012746), but neither matched 5-versus-0 nor 10-versus-0 mg/L comparison remained significant after Holm adjustment; we therefore did not interpret this isolated result as evidence of a distinct mTOR mechanism. γ-H2AX showed a marked directional increase across the MDA-MB-231 series (mean 340.17% at 5 mg/L and 786.03% at 10 mg/L relative to the within-experiment control), but the repeated-measures ANOVA did not reach statistical significance (*p* = 0.124629).

These replicate-level data support the use of BT-474 and MDA-MB-231 as comparison models rather than as independent reproductions of the coordinated MCF-7 ER/PI3K-AKT/FoxO response. The magnitude and pattern of the measured protein-marker changes were more variable or qualitatively different across the comparison cell lines.

### 2.6. Reduced Puromycin Labeling and MG132 Testing Inform Protein-Homeostasis Interpretation

Puromycin labeling was visibly reduced after o-GQD exposure, consistent with reduced nascent protein synthesis. The separate puromycin-only control confirmed assay detectability. Because replicate-complete puromycin total-lane densitometry was unavailable, this remains a qualitative observation.

**Figure 5:**
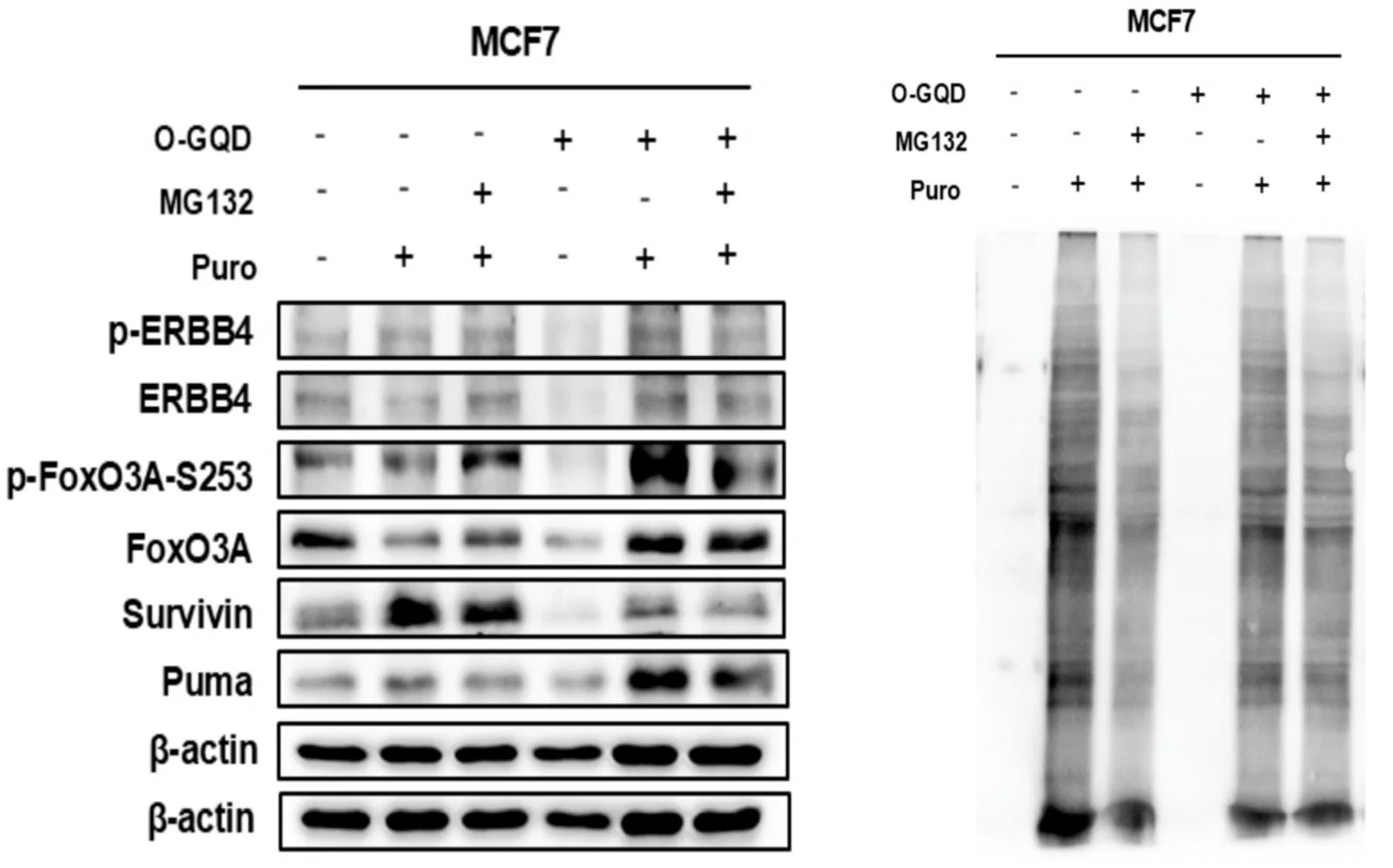
MG132/puromycin treatment series in MCF-7 cells. Protein-marker values are from three independent biological experiments and are shown as mean ± SD with individual replicate values. For the four puromycin-present conditions, o-GQD and MG132 effects were assessed using a two-factor repeated-measures ANOVA. No o-GQD × MG132 interaction reached statistical significance for the four quantified endpoints (interaction *p* = 0.1919–0.3190). A secondary matched control-versus-o-GQD comparison showed a marked Survivin reduction (raw *p* = 0.0232) that did not remain significant after Holm correction across the four protein endpoints (adjusted *p* = 0.0929). The separate puromycin-only control verified assay labeling/detection detectability.

We tested MG132 reversal of selected protein-marker changes in three independent biological experiments measuring p-ErbB4/ErbB4, p-FoxO3a (S253)/FoxO3a, Survivin/β-actin, and PUMA/β-actin (Figure 5). Across the four puromycin-present conditions, two-factor repeated-measures ANOVA showed no statistically significant o-GQD × MG132 interaction for any endpoint (interaction *p* = 0.1919–0.3190; n = 3). Survivin decreased directionally in the o-GQD-only condition across all three experiments; the matched control-versus-o-GQD contrast did not remain significant after correction across the four quantified protein endpoints (raw *p* = 0.0232; Holm-adjusted *p* = 0.0929). MG132 did not consistently restore the selected markers, arguing against a simple proteasome-dependent explanation for these marker changes.

### 2.7. Working Model: Convergent Evidence for a Coordinated MCF-7 Response

**Figure 6:**
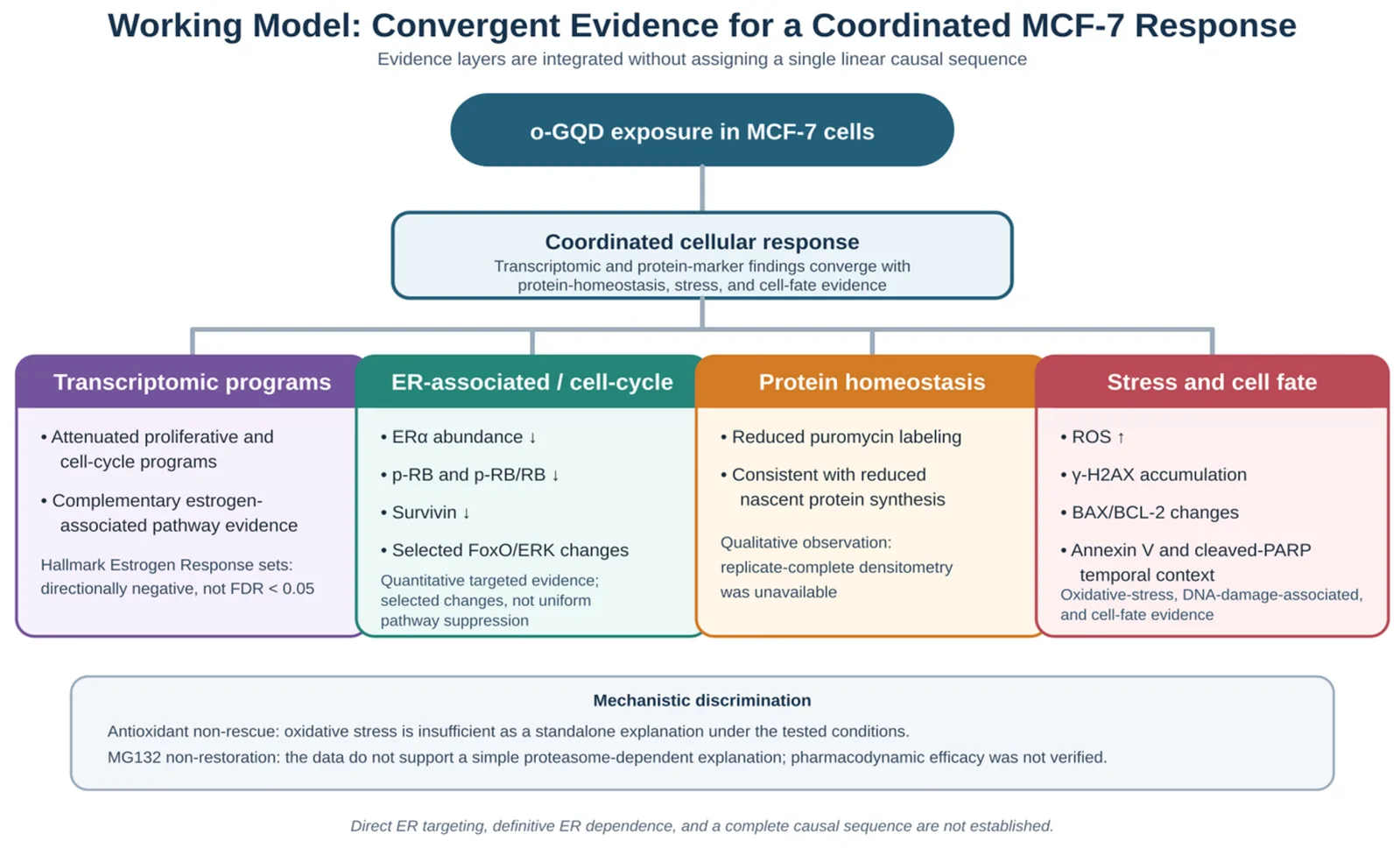
Working model of the coordinated MCF-7 response to o-GQD. Transcriptomic evidence of attenuated proliferative and estrogen-associated programs converges with selected ER-associated/cell-cycle protein-marker changes and reduced puromycin labeling consistent with reduced nascent protein synthesis. Protein-homeostasis, ROS/stress, γ-H2AX, and apoptosis-related findings form interconnected evidence layers.

The transcriptomic, protein-marker, puromycin, oxidative-stress, DNA-damage-associated, and cell-fate findings converge in a working model of the MCF-7 response (Figure 6). Attenuated proliferative and estrogen-associated programs align with ER-associated/cell-cycle protein-marker changes and reduced puromycin labeling consistent with reduced nascent protein synthesis. ROS, NRF2, γ-H2AX, BAX/BCL-2, and p53-related programs add stress-, DNA-damage-associated-, and apoptosis-related dimensions to this interconnected response.

## 3. Discussion

### 3.1. Overview of Findings

RNA-seq programs and targeted protein-marker findings converge on coordinated alterations in estrogen-associated survival and cell-cycle biology in MCF-7 cells. Attenuated proliferative and estrogen-associated transcriptomic programs were accompanied by quantitative changes in ERα, RB, Survivin, selected FoxO/ERK readouts, and γ-H2AX, together with reduced puromycin labeling consistent with reduced nascent protein synthesis. ROS accumulation, NRF2 reduction, DNA-damage-associated changes, and apoptosis-related responses complete this integrated biological picture.

Antioxidant co-treatment and MG132 testing evaluated oxidative stress and proteasomal degradation as competing standalone explanations; neither produced a consistent rescue pattern under the tested conditions. The weaker or different responses in BT-474 and MDA-MB-231 cells show that cellular context shapes the magnitude and pattern of the response.

### 3.2. Western-Blot Quantification Supports ERα Reduction and Strong RB/Survival-Marker Responses

ERα can interact with PI3K-AKT signaling, and AKT-mediated phosphorylation regulates FOXO-family activity [2,9,10]. FOXO-related signaling can in turn affect survival mediators including Survivin and PUMA [11,12]. For Figure 3, direct protein abundance and phospho/total ratios were analyzed separately according to the matched experimental design.

Separating direct abundance from phospho/total ratios clarified the protein response. Total ERα abundance decreased strongly even though the p-ERα/ERα ratio was not consistently reduced, because both phosphorylated and total ERα changed. Direct p-RB likewise showed a much larger and more consistent decrease than total RB, supporting marked RB dephosphorylation. Survivin was also strongly reduced across four matched experiments. p-FoxO1/FKHR-S256 and total FoxO3A decreased, while several other phospho/total ratios were more variable. ER-associated cell-cycle control and FOXO signaling are also relevant to endocrine-response and resistance biology in breast cancer [3,13]. Together, these markers define selected ER-associated, FoxO/cell-cycle, and survival-related changes within the broader response.

The quantitative analysis also supported a consistent decrease in p-ERK/ERK and a marked directional increase in γ-H2AX. BCL-2 and BAX showed biologically coherent but more variable directional changes. These targeted protein-level findings converge with the transcriptomic attenuation of proliferative, cell-cycle, and complementary estrogen-associated programs, linking program-level and marker-level evidence.

The focused n = 2 Western-blot time course adds temporal context to these endpoint findings. ERα abundance was already reduced at 24–48 h, whereas pronounced cleaved-PARP accumulation appeared at 72 h. BCL-2 decreased progressively and BAX remained above baseline. By contrast, p-AKT/AKT and p-FoxO3A-S253/FoxO3A increased at several later time points, indicating dynamic or adaptive signaling rather than a simple monotonic suppression sequence.

### 3.3. Reduced Puromycin Labeling and Proteasome Inhibition

Reduced puromycin labeling was observed after o-GQD exposure, consistent with reduced nascent protein synthesis. In the three-experiment MG132/puromycin series, no statistically significant o-GQD × MG132 interaction was detected across the four quantified protein-marker endpoints. Survivin showed the clearest directional response, decreasing in the o-GQD-only condition, although its matched contrast did not remain significant after multiplicity correction.

Together, these findings identify reduced nascent protein synthesis as a protein-homeostasis component of the MCF-7 response and argue against a simple proteasome-dependent explanation for the selected marker changes: the absence of a significant o-GQD × MG132 interaction across all four endpoints distinguishes the response from proteasome-mediated marker loss. Two boundaries apply: the puromycin labeling result is qualitative because replicate-complete densitometry is unavailable, and MG132 pharmacodynamic efficacy was not independently verified in the principal Figure 5 series, so a proteasomal contribution cannot be definitively excluded [14,15]. Replicate-complete puromycin quantification and validated proteasome-inhibition controls would resolve the relative contributions of synthesis and degradation.

### 3.4. ROS and DNA-Damage-Associated Responses

An increased ROS-positive fraction and lower NRF2 abundance accompanied o-GQD exposure. Neither NAC nor MitoTEMPO rescued the cytotoxic phenotype under the tested conditions, indicating that ROS is a component of the response but is insufficient as a standalone explanation. Supplementary Figure S1 shows an ROS response at 24 h, preceding the principal 72 h endpoint. The two-experiment ROS/Annexin V time course adds descriptive timing for oxidative-stress and apoptosis-associated readouts. These focused observations provide temporal context; they do not resolve the initiating event or the ordering of the full response.

γ-H2AX accumulation and the accompanying BAX and survival-marker changes were consistent with DNA-damage-associated and apoptosis-related responses. These findings connect the oxidative-stress and cell-fate evidence to the central signaling and protein-homeostasis response.

### 3.5. Biological and Mechanistic Interpretation

The defined o-GQD preparation served as a controlled perturbation for resolving the molecular response of MCF-7 cells. RNA sequencing showed attenuation of proliferative and cell-cycle programs, with complementary estrogen-associated pathway evidence. At the protein level, reduced ERα abundance, strong RB dephosphorylation, Survivin suppression, and selected FoxO/ERK changes converged with these program-level findings. This convergence places estrogen-associated survival and cell-cycle regulation at the center of the response while preserving the distinction between transcriptomic programs and targeted protein markers.

ROS accumulation, γ-H2AX induction, BAX/BCL-2 changes, and the descriptive Annexin V/time-course evidence connect cellular stress with DNA-damage-associated and cell-fate responses. Reduced puromycin labeling consistent with reduced nascent protein synthesis adds a protein-homeostasis component. Together, these layers support an interconnected cellular response, with antioxidant and MG132 experiments testing competing standalone explanations.

### 3.6. Limitations and Future Directions

These findings define the response to a specific o-GQD preparation principally in the MCF-7 in vitro model. BT-474 and MDA-MB-231 provide limited comparison-cell-line context; broad ER-positive selectivity and generalizability remain to be tested. In our prior o-GQD study, T-47D cells showed an o-GQD-associated reduction in viability and a pronounced Annexin V-positive apoptotic response [5], which extends the phenotype beyond MCF-7 but did not reproduce the detailed ER/PI3K-AKT/FoxO protein-marker analysis performed here; the published T-47D evidence therefore provides phenotype context rather than mechanistic replication or evidence of definitive ER dependence. A non-malignant mammary epithelial comparison such as MCF-10A was not included. Several targeted endpoints have small biological n. The study did not include direct ER target-engagement or functional ER-dependence experiments; direct ER targeting and functional ER dependence remain unestablished.

RNA sequencing and the principal mechanistic analyses focused on the 72 h endpoint. Supplementary Figure S1 adds a 24 h ROS observation; the ROS/Annexin V and Western-blot time courses each comprise n = 2 descriptive experiments, and a replicate-complete matched time-by-dose matrix is unavailable. Puromycin evidence is qualitative because replicate-complete quantitative densitometry is unavailable. MG132 efficacy and NAC/MitoTEMPO pharmacodynamic activity were not independently validated. Although the DHE readout was spectrally separated from intrinsic o-GQD autofluorescence, cell-free controls to assess other optical or probe-chemistry interference were not performed. Temporal ordering and the relative contributions of protein synthesis, degradation, and oxidative mechanisms therefore remain open questions.

## 4. Materials and Methods

### 4.1. Preparation and Characterization of the Graphene Material

We used a polyethylene glycol (PEG)-coated graphene quantum dot synthesized through a one-pot hydrothermal process. We used o-phenylenediamine (OPD) as the nitrogen and carbon source and conducted the reaction at 300 °C in the presence of PEG [16], producing a heterogeneous mixture of GQDs with different molecular weights and surface functionalities. We isolated a nitrogen- and oxygen-containing fraction structurally related to OPD and refer to it here as o-GQD. Because this preparation was previously associated with delayed cell-cycle progression and apoptosis-related responses in breast cancer cells [5], we used the defined preparation as a fixed material-based perturbation to examine the accompanying transcriptomic, signaling, stress, and protein-synthesis responses. The present experiments were designed to resolve the cellular response rather than to optimize the material.

Surface chemistry was characterized by X-ray photoelectron spectroscopy (XPS) and Fourier-transform infrared spectroscopy (FT-IR; Nicolet spectrometer), with high-resolution C 1s, N 1s, and O 1s peak fitting performed using an optimization algorithm developed in-house by the collaborating laboratory. The survey spectra compared pristine and PEG-modified o-GQD samples, and the FT-IR spectra compared pristine and PEG-coated o-GQD samples (Supplementary Figure S4). The XPS data indicated substantial surface oxidation and nitrogen-containing functionality, with oxidation and amidation levels recorded as 48.4 (O/C ratio) and 4.6 at.% (N/C ratio), respectively (Fison VG ESCA-210, Fison Instruments Ltd., Glasgow, UK). The FT-IR spectra showed features in the 1200–1300 cm^−1^ region consistent with C-O stretching, a band near 1710 cm^−1^ attributable to C=N and/or C=O surface functionality, and a broad band near 3250–3400 cm^−1^ associated with adsorbed water and/or N-H groups. These findings support describing the surface as PEG-modified or PEG-associated; covalent bonding between PEG and the o-GQD core was not directly established. Particle morphology and size distribution were examined by transmission electron microscopy (FEI Talos F200s, Thermo Fisher Scientific, Waltham, MA, USA, 200 kV) and dynamic light scattering (DLS) measured in distilled water. Quantitative DLS mean/Z-average and PDI, zeta potential, and colloidal stability in culture medium were not available for the batch used in the biological experiments and are therefore not reported.

### 4.2. RNA Sequencing and Differential Expression Analysis

We treated MCF-7 cells with 10 mg/L o-GQD for 72 h and used time-matched untreated cells as controls, with three independent biological replicates per group and six samples in total. We isolated total RNA using NucleoZOL (Macherey-Nagel GmbH & Co. KG, Düren, Germany) according to the manufacturer’s protocol, assessed RNA integrity with an Agilent 2100 Bioanalyzer (Agilent, Santa Clara, CA, USA), and processed only samples with an RNA integrity number greater than 8.0 for sequencing.

We prepared strand-specific mRNA libraries with the Illumina Stranded mRNA Prep Ligation Kit. Taiwan Genomic Industry Alliance Inc. (project TS230825003) performed sequencing on an Illumina NovaSeq 6000 platform (Illumina, San Diego, CA, USA) using 150 bp paired-end reads, generating approximately 20 million reads per sample. We assessed read quality using FastQC [17], trimmed adapters using Trimmomatic [18], aligned clean reads to GRCh38 with STAR v2.7.10a [19], generated gene-level counts with featureCounts [20], and analyzed differential expression using DESeq2 v1.34.0 [21].

### 4.3. Gene-Set Enrichment and Pathway Analyses

We ranked genes using the sign of the DESeq2 log2 fold change multiplied by −log10(adjusted *p* value + 1 × 10^−300^) and converted Ensembl identifiers to Entrez identifiers. We performed ranked-list gene-set enrichment analysis for the MSigDB Hallmark collection [22,23] and used the same ranked evidence framework for complementary Gene Ontology, KEGG, and Reactome analyses. This design allowed us to interpret Hallmark, pathway-database, and ontology findings as separate evidence layers.

We set the minimum and maximum gene-set sizes to 15 and 1000, respectively, adjusted *p* values using the Benjamini–Hochberg method, and applied an FDR q value below 0.05 as the significance threshold. We interpreted normalized enrichment scores according to enrichment toward the upregulated or downregulated end of the ranked list. We summarized the results using enrichment plots, dot or bubble plots, and leading-edge gene lists and compared these program-level findings with the protein-level results.

### 4.4. Determination of Experimental Concentrations Using MTT Assay

In at least three independent MTT dose–response experiments, we treated MCF-7 cells with 0–40 mg/L o-GQD for 72 h. The estimated half-maximal inhibitory concentrations ranged from 19 to 28 mg/L across independent experimental runs. Working concentrations of 5 and 10 mg/L were selected below the estimated IC50 range. In pilot observations, apoptosis-related and Western blot changes were less readily detectable at 48 h and more apparent at 72 h; we consequently used 72 h as the principal endpoint for characterizing the developed molecular response.

### 4.5. Western Blotting

We lysed cells in RIPA buffer supplemented with protease and phosphatase inhibitors and measured protein concentrations using a BCA assay. We separated equal protein amounts (20–30 μg) by SDS-PAGE, transferred them to PVDF membranes, and incubated the blocked membranes with antibodies against the selected ER-, PI3K-AKT-FoxO-, cell-cycle-, stress-, apoptosis-, and protein-synthesis-related targets. We used β-actin as the loading control where indicated, developed signals by enhanced chemiluminescence, imaged them with a Fusion FX7 system, and quantified them using ImageJ (Version 1.54) [24]. We performed Western blot experiments in at least three independent biological replicates for the principal endpoint experiments. For Figure 3, values were normalized within each experiment to the untreated control. Biological n is marker-specific (generally n = 3, with n = 4 or 5 for selected endpoints), and direct protein abundance and phospho/total ratios were treated as distinct endpoints. The Figure 4 comparison-cell-line analysis and Figure 5 protein-marker analysis are each based on three independent biological experiments. A focused MCF-7 Western-blot time course was additionally performed in two independent biological experiments after exposure to 10 mg/L o-GQD for 0, 24, 48, or 72 h. This n = 2 series was normalized within the experiment and is presented descriptively.

### 4.6. Intracellular ROS Measurement

We measured intracellular ROS using the DHE-based Muse Oxidative Stress Kit (MCH100111; Merck KGaA, Darmstadt, Germany) and a Muse Cell Analyzer (Merck KGaA, Darmstadt, Germany). The principal Figure 2 analysis was performed after treatment with 0, 5, or 10 mg/L o-GQD for 72 h. Supplementary Figure S1 additionally reports ROS-positive and ROS-negative populations after 24 h of exposure to 5 or 10 mg/L o-GQD, with or without 20 μM MitoTEMPO. For antioxidant intervention, we administered N-acetylcysteine (NAC; 3–20 mM) or MitoTEMPO (20 μM) [25,26] before o-GQD exposure under the tested conditions. We processed the cells according to the assay workflow and evaluated ROS-positive and ROS-negative populations. The DHE readout was spectrally separated from the reported intrinsic o-GQD autofluorescence region; this reduces concern about direct spectral overlap but does not exclude all optical or probe-chemistry interference. These experiments tested whether antioxidant intervention rescued the observed response. A focused time-course series of ROS and Annexin V readouts was additionally performed in two independent biological experiments to provide supplementary temporal context. Both experiments are presented descriptively.

### 4.7. Puromycin Incorporation and Figure 5 Replication

We assessed puromycin labeling by anti-puromycin immunoblotting as a readout of nascent protein synthesis. The Figure 5 MG132/puromycin treatment series was performed in three independent biological experiments. Protein-marker signals were normalized to the corresponding β-actin denominator and then to the untreated control within each experiment; phosphorylation ratios were calculated from the corresponding normalized phospho- and total-protein values. Equal protein amounts (20–30 µg per lane) were loaded as described above. A separate single puromycin-only labeling control was used only to establish that labeling/detection was functional; it was not treated as a biological replicate or as a proof of pathway-specific translational suppression. Replicate-complete puromycin total-lane densitometry was not available.

### 4.8. Statistical Analysis

Within each marker, the 0, 5, and 10 mg/L values from the same biological experiment were analyzed using one-way repeated-measures ANOVA as the primary endpoint-specific analysis. Matched 5-versus-0 and 10-versus-0 mg/L secondary contrasts used two-sided paired tests with Holm adjustment within each endpoint. Biological n was marker-specific (n = 3–5). Because these proteins represent targeted mechanistic readouts rather than an untargeted screening panel, across-endpoint Benjamini–Hochberg correction was retained only as a conservative family-level sensitivity analysis rather than used as the sole criterion for biological interpretation. Friedman tests were likewise used only as a small-sample nonparametric sensitivity analysis. Direct protein abundance and phospho/total ratios were analyzed separately, and results were interpreted together with effect magnitude, direction across biological replicates, and assay context.

For the Figure 4 comparison-cell-line analysis, BT-474 and MDA-MB-231 cells were evaluated in three independent biological experiments at 0, 5, and 10 mg/L o-GQD. Within each experiment, quantified values were expressed relative to the corresponding untreated control. For each marker, the three dose conditions were analyzed as matched repeated measures using one-way repeated-measures ANOVA. Where dose-versus-control post hoc comparisons were examined, the 5-versus-0 and 10-versus-0 mg/L paired comparisons were adjusted by the Holm method.

For the Figure 5 protein-marker series, three independent biological experiments were analyzed as matched repeated measures. The four puromycin-present conditions were treated as a 2 × 2 repeated-measures design with o-GQD (absent/present) and MG132 (absent/present) as within-experiment factors. Endpoint-specific o-GQD, MG132, and interaction effects were assessed by two-factor repeated-measures ANOVA. Where secondary matched contrasts were reported outside this factorial subset, paired two-sided tests were used with Holm adjustment across the four quantified protein endpoints. Exact *p* values are reported in the corresponding results.

The focused ROS/Annexin V time-course series comprised two independent experiments and was therefore summarized descriptively without formal inferential statistics. The focused Western-blot time course likewise comprised two independent experiments and was summarized descriptively without formal inferential statistics.

## 5. Conclusions

Exposure to a defined o-GQD preparation produces a coordinated response in MCF-7 cells involving attenuation of proliferative and estrogen-associated transcriptional programs, RB/cell-cycle regulation, and selected ER-associated and survival-related protein markers, together with oxidative-stress and DNA-damage-associated responses. Convergence between transcriptomic and protein-level findings identifies these changes as components of an integrated cellular response. Reduced puromycin labeling consistent with reduced nascent protein synthesis adds a protein-homeostasis dimension.

Antioxidant co-treatment did not rescue the cytotoxic phenotype, and MG132 did not consistently restore the selected protein-marker changes, narrowing oxidative stress and proteasomal degradation as standalone explanations. The focused time courses provide descriptive temporal separation among selected signaling and apoptosis-associated readouts.

Together, these results support a model in which o-GQD exposure disrupts interconnected estrogen-associated survival, cell-cycle, and protein-homeostasis programs in MCF-7 cells while engaging cellular stress and cell-fate responses. This integrated framework advances the mechanistic interpretation of the previously observed growth-inhibitory phenotype.

## Data Availability

The original contributions presented in this study are included in the article/Supplementary Material. Further inquiries can be directed to the corresponding author(s). The raw and processed RNA-sequencing data generated in this study have been deposited in the NCBI Gene Expression Omnibus (GEO) under accession number GSE346126.

## Supplementary Materials

The following supporting information can be downloaded at: https://www.mdpi.com/article/10.3390/ijms27209211/s1.
