# Supplementary material for: Integrated Transcriptomic and Biochemical Analyses Reveal Coordinated Suppression of ER-Associated Survival Signaling and Activation of ROS-Mediated Stress Responses by Oxidized Graphene Quantum Dots in Estrogen Receptor-Positive Breast Cancer
Source: Int J Mol Sci. 2026 Oct 16;27(20):9211. doi: 10.3390/ijms27209211 (PMC13705060; doi:10.3390/ijms27209211)
Supplement: Supplementary file 1 [file ijms-27-09211-s001.zip › ijms-4503693-supplenmetary.pdf]

Supplementary Materials: The following supporting information is included in this manuscript: Figure S1, MitoTEMPO intervention in the MCF-7 ROS assay; Figure S2, focused time-course ROS and Annexin V analysis; Figure S3, focused time-course Western-blot analysis; Figure S4, XPS and FT-IR characterization of the o-GQD preparation; Table S1, estrogen-response gene-set enrichment statistics with leading-edge genes.

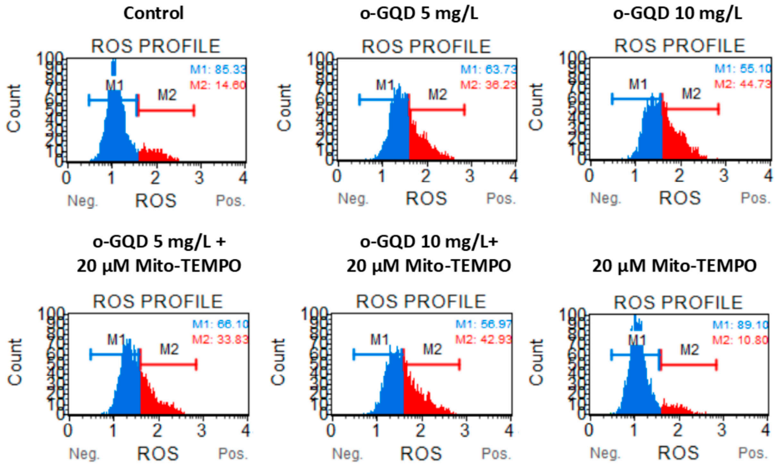

Figure S1. Representative Muse Cell Analyzer histograms show ROS-positive and ROS-negative populations after treatment with 5 or 10 mg/L o-GQD for 24 h, with or without 20 μM MitoTEMPO. MitoTEMPO did not rescue the phenotype under the tested conditions.

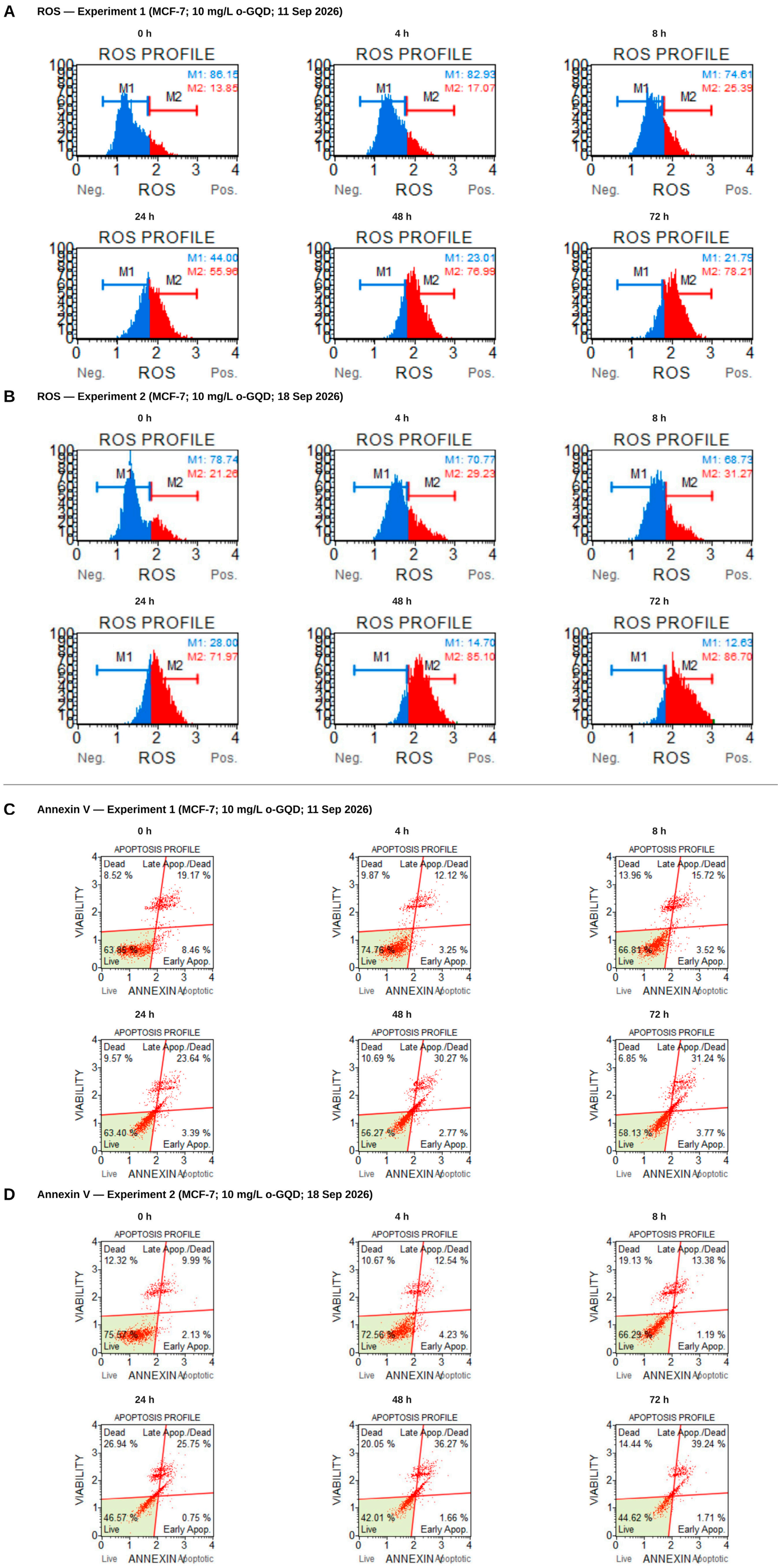

Figure S2. Focused time-course ROS and Annexin V analysis in MCF-7 cells. Two independent biological experiments (n = 2) are shown descriptively to provide temporal context for oxidative-stress and apoptosis-associated readouts; no formal inferential statistics were applied.

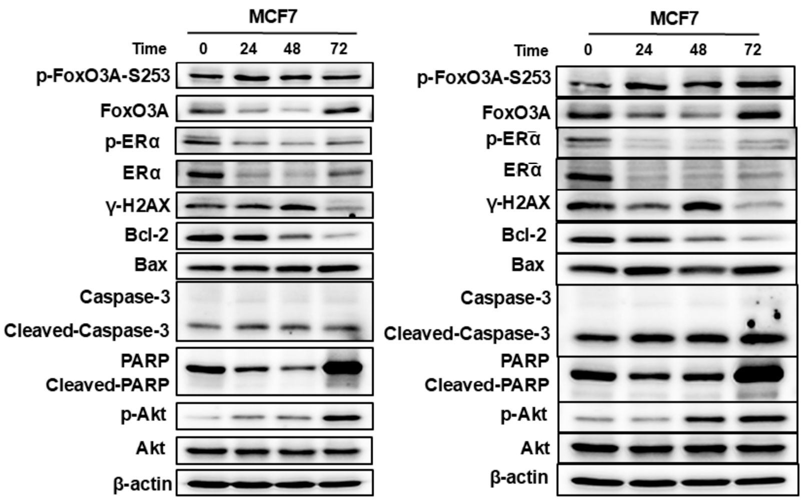

Figure S3. Focused time-course Western-blot analysis in MCF-7 cells exposed to 10 mg/L o-GQD for 0, 24, 48, or 72 h. Two independent biological experiments (n = 2) are shown descriptively. ER*α* abundance decreased by 24–48 h in both experiments, BCL-2 decreased progressively, BAX remained above baseline, and marked cleaved-PARP accumulation was observed at 72 h. p-AKT/AKT and p-FoxO3A-S253/FoxO3A showed non-monotonic or adaptive increases rather than uniform temporal suppression.

(a)

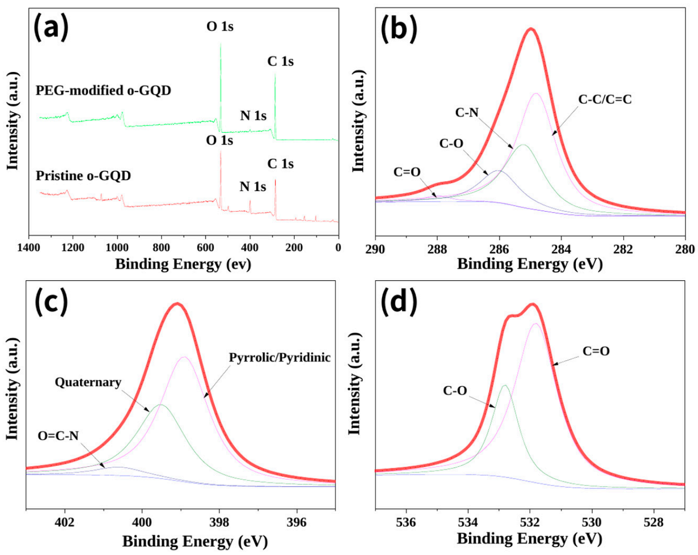

(b)

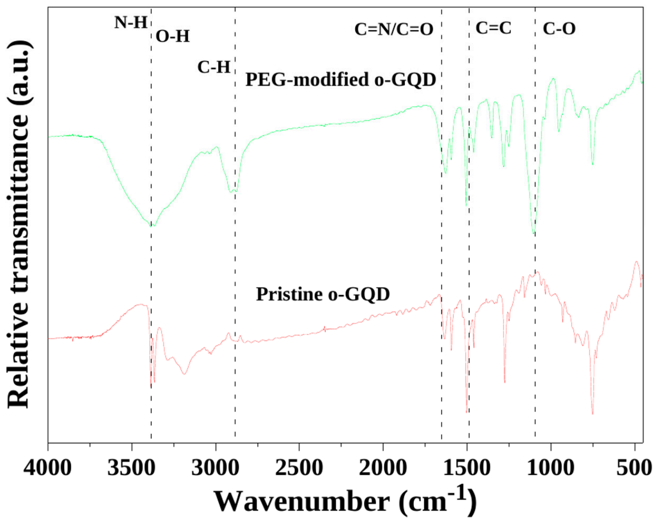

Figure S4. Physicochemical characterization of the o-GQD preparation. (a) Survey-scan XPS of pristine and PEG-modified o-GQD samples, and high-resolution XPS spectra of the o-GQD sample: C 1s, N 1s, and O 1s. (b) FT-IR spectra of pristine o-GQD and PEG-coated o-GQD samples. The data support a PEG-modified, oxygen- and nitrogen-functionalized surface; quantitative DLS mean/Z-average and PDI, zeta potential.
